# The Complex Conformational Dynamics of Neuronal Calcium Sensor-1: A Single Molecule Perspective

**DOI:** 10.3389/fnmol.2018.00468

**Published:** 2018-12-17

**Authors:** Dhawal Choudhary, Birthe B. Kragelund, Pétur O. Heidarsson, Ciro Cecconi

**Affiliations:** ^1^Department of Physics, Informatics and Mathematics, University of Modena and Reggio Emilia, Modena, Italy; ^2^Center S3, CNR Institute Nanoscience, Modena, Italy; ^3^Structural Biology and NMR Laboratory, Department of Biology, University of Copenhagen, Copenhagen, Denmark; ^4^Department of Biochemistry, University of Zurich, Zurich, Switzerland

**Keywords:** NCS-1, protein folding and misfolding, calcium binding, optical tweezers, single molecule studies

## Abstract

The human neuronal calcium sensor-1 (NCS-1) is a multispecific two-domain EF-hand protein expressed predominantly in neurons and is a member of the NCS protein family. Structure-function relationships of NCS-1 have been extensively studied showing that conformational dynamics linked to diverse ion-binding is important to its function. NCS-1 transduces Ca^2+^ changes in neurons and is linked to a wide range of neuronal functions such as regulation of neurotransmitter release, voltage-gated Ca^2+^ channels and neuronal outgrowth. Defective NCS-1 can be deleterious to cells and has been linked to serious neuronal disorders like autism. Here, we review recent studies describing at the single molecule level the structural and mechanistic details of the folding and misfolding processes of the non-myristoylated NCS-1. By manipulating one molecule at a time with optical tweezers, the conformational equilibria of the Ca^2+^-bound, Mg^2+^-bound and apo states of NCS-1 were investigated revealing a complex folding mechanism underlain by a rugged and multidimensional energy landscape. The molecular rearrangements that NCS-1 undergoes to transit from one conformation to another and the energetics of these reactions are tightly regulated by the binding of divalent ions (Ca^2+^ and Mg^2+^) to its EF-hands. At pathologically high Ca^2+^ concentrations the protein sometimes follows non-productive misfolding pathways leading to kinetically trapped and potentially harmful misfolded conformations. We discuss the significance of these misfolding events as well as the role of inter-domain interactions in shaping the energy landscape and ultimately the biological function of NCS-1. The conformational equilibria of NCS-1 are also compared to those of calmodulin (CaM) and differences and similarities in the behavior of these proteins are rationalized in terms of structural properties.

## NCS-1 Is a Multi-Functional, Two-Domain Protein

Calcium ion (Ca^2+^) signaling is crucial for neurotransmitter release and is intrinsic for neuronal functions. Calcium signaling is mediated by calcium binding proteins that sense changes in cellular concentration of Ca^2+^ ions and respond by interacting with downstream regulatory targets, further cascading the signal. One example of such calcium sensor proteins is the family of neuronal calcium sensors (NCSs), which are expressed primarily in neurons and photoreceptor cells (Weiss et al., 2010; Reyes-Bermudez et al., 2012). These proteins respond to changes in Ca^2+^ concentration through conformational changes that allow them to bind diverse protein partners (Burgoyne and Haynes, 2012). The NCS family has 15 highly-conserved members in mammals and these proteins have numerous functions (Burgoyne, 2007). NCS-1 is the primordial member of the family and is the most widely expressed. It has been shown to have roles in an array of cellular processes, such as regulation of Ca^2+^- (N and P/Q type) and K^+^ (Kv4)-channels (Weiss et al., 2000; Nakamura et al., 2001; Guo et al., 2002; Tsujimoto et al., 2002), phosphodiesterase activity (Burgoyne et al., 2004), membrane trafficking (McFerran et al., 1999), and the direct regulation of the dopamine D2 receptor and the GRK2 kinase (Kabbani et al., 2002). A connection between defective NCS-1 and neurodegenerative disorders like schizophrenia, autism and bipolar disorder has also been suggested (Kabbani et al., 2002; Koh et al., 2003; Bai et al., 2004; Handley et al., 2010; Pavlowsky et al., 2010). It was observed that in comparison to healthy individuals, patients suffering from schizophrenia had upregulated levels of NCS-1 in the prefrontal cortex, which may explain their reduced prefrontal cortex activity (Kabbani et al., 2002; Koh et al., 2003). Also, NCS-1 has been attributed to the regulation of synaptic activity as it directly interacts with D2 receptors and it is plausible that defective NCS-1 may lead to impairment of cognitive functions and mental retardation (Burgoyne, 2007).

**Figure 1:**
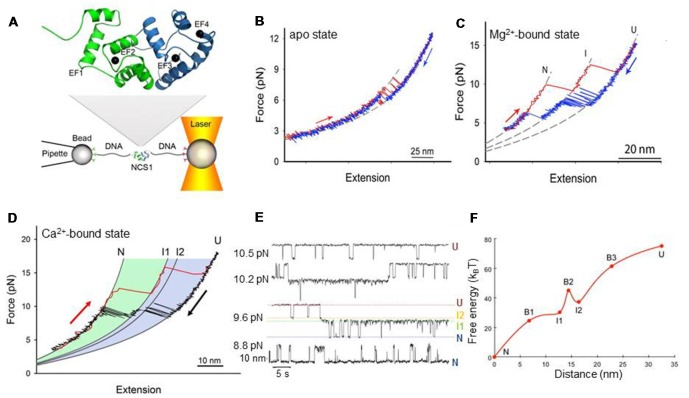
Mechanical manipulation of a single non-myristoylated neuronal calcium sensor-1 (NCS-1) molecule. **(A)** Schematic representation of the optical tweezers setup. A NCS-1 molecule is tethered to two polystyrene beads by means of molecular handles (~500 bp DNA molecules) that function as spacers to avoid unspecific interaction between the tethering surfaces (Cecconi et al., 2005). One bead is held in an optical trap, while the other is held at the end of a pipette by suction. During the experiment the protein is stretched and relaxed by moving the pipette relative to the optical trap, and the applied force and the molecular extension are measured as described in Smith et al. (2003). The inset shows the NMR structure of NCS-1 (PDB code 2LCP), where the C- and N-domains are shown in blue and green, respectively. **(B)** Force vs. extension cycle obtained by stretching (red trace) and relaxing (blue trace) NCS-1 in the absence of divalent ions. **(C)** Force vs. extension cycle obtained by stretching (red trace) and relaxing (blue trace) NCS-1 in the presence of 10 mM Mg^2+^. **(D)** Mechanical manipulation of the Ca^2+^-bound state of NCS-1. During stretching (red trace) the N-domain unfolds at ~13 pN and the C-domain at ~16 pN. During relaxation (black trace), U folds into N through a four-state process (U > I2 > I1 > N) coordinated by calcium binding. **(E)** Extension vs. time traces acquired at different constant forces showing the Ca^2+^-bound state of NCS-1 fluctuating at equilibrium between N, I1, I2 and U. The population probability of the different states can be modulated by force. Analysis of these experimental data with the Hidden Markov Model allows for the characterization of the energy landscape of the protein in terms of activation energy barriers, separating the different molecular states, and positions of the transition states along the reaction coordinate (Rabiner, 1989; Chodera et al., 2011). **(F)** Energy landscape of the Ca^2+^-bound state of NCS-1 at zero force. The transition states of the different folding reactions are indicated with the letter B. The activation barriers separating the different molecular states were calculated using a pre-exponential factor of 1.2 × 10^−4^ Hz (Gebhardt et al., 2010). Panels adapted from Heidarsson et al. (2013b) and Naqvi et al. (2015).

All NCS proteins are composed of ~200 residues organized in a compact and globular structure, have either an N-terminal myristoylation or palmitoylation site (Pongs et al., 1993; Rivosecchi et al., 1994; Tsujimoto et al., 2002; Burgoyne, 2007), and bind calcium through EF-hand motifs. NCS-1 is an all-helical 190 residue protein organized in two ~100 a.a. domains, each containing a pair of EF-hands; the N-domain EF1 and EF2, and the C-domain EF3 and EF4 (Figure 1A; Heidarsson et al., 2012a). Due to a conserved Cys/Pro substitution in EF1, NCS-1 is only capable of binding three calcium ions (Aravind et al., 2008; Grabarek, 2011), a trait of all NCS family members except for recoverin and KChIP1 who have only two active EF-hands (Scannevin et al., 2004; Zhou et al., 2004). In NCS-1, EF2 and EF3 are structural sites as they can bind to both Ca^2+^ and Mg^2+^, whereas EF4 is a regulatory site only binding Ca^2+^ and with lower affinity (Aravind et al., 2008). A few recent high-resolution structures are available for NCS-1, which all show excellent agreement regarding the general tertiary structure, the relative orientation of the two domains, and the locations of α-helices (Ames et al., 2000; Bourne et al., 2001; Strahl et al., 2007; Heidarsson et al., 2012a). An NMR study on NCS-1 suggested that independent movements of the N- and C-domains can occur, which may help accommodating different ligands in the binding pocket, and that the last 15 residues of the protein are disordered yet crucial for conformational stability, undergoing conformational exchange that may regulate ligand binding (Heidarsson et al., 2012a). The equilibrium unfolding of NCS-1 has been well studied using guanidinium chloride (GdmCl) denaturation and shows that the protein unfolds through two separate unfolding transitions, corresponding to the sequential unfolding of the N- (unfolding at 3.1 M GdmCl with ΔG ~35 KJ mol^−1^) and C-domain (unfolding at 4.6 M GdmCl with ΔG ~38 KJ mol^−1^; Aravind et al., 2008; Heidarsson et al., 2012a). While the structure, thermodynamics, and biological functions of NCS-1 have been elucidated in detail with different techniques, the folding and misfolding mechanisms of this protein have long remained elusive. This information gap has recently been bridged by single-molecule optical tweezers studies.

## A Single Molecule Perspective on Protein Folding Using Direct Mechanical Manipulation

Protein folding, especially in the case of large multi-domain proteins, is a complex process encompassing many concerted events such as breaking of non-native contacts, formation of native interactions and changes in conformation and dynamics (Batey et al., 2008; Borgia et al., 2015; Jahn et al., 2016). In large multi-domain proteins different domains can fold and unfold *via* different pathways either independently or dependently and intermediate states can be populated on the way to a native conformation. Their folding and unfolding processes are also dependent on the crosstalk between the domains and on internal friction, brought about by a frustrated search for inter-domain contacts between partially formed domains. Traditional methods such as X-ray crystallography, NMR and optical spectroscopies have been extraordinarily powerful to decipher the structure and folding characteristics of proteins. However, the ensemble nature of the signal provided by such techniques can blur the diversity of folding trajectories and short lived populations, making it difficult to gauge the complicated sequence of events that characterize the folding mechanism of proteins and other biomolecules.

Single molecule force spectroscopy using optical tweezers enables direct mechanical manipulation of individual molecules and the detailed characterization of their conformational equilibria under tension (Cecconi et al., 2005; Shank et al., 2010; Yu et al., 2012; Xu and Springer, 2013; Alemany et al., 2016; Zhang, 2017; Jahn et al., 2018; Wruck et al., 2018). In these experiments, a single molecule is tethered between two polystyrene beads using DNA molecular handles and then is stretched and relaxed by changing the distance between the tethering surfaces (Figure 1A; Heidarsson et al., 2013a). Under tension, the unfolding and refolding of a protein is accompanied by changes in the extension of the molecule, giving rise to discontinuities (rips) in the recorded force traces. A molecule can be stretched and relaxed at constant speed (Heidarsson et al., 2012b; Caldarini et al., 2014), or it can be kept at a specific force through a force feedback mechanism and observed to fluctuate between different molecular conformations (Heidarsson et al., 2013b). A careful analysis of the experimental data and the use of advanced statistical methods allow a detailed characterization of the folding and, possibly, misfolding processes of the molecule and the reconstruction of its energy landscape (Stigler et al., 2011). An important limitation of optical tweezers to keep in mind is that they describe three-dimensional molecular processes, such as the folding of a protein, along a single reaction coordinate defined by the points of force application, namely the residues to which the DNA handles are attached. As a consequence, during the mechanical manipulation of a molecule, all structural variations that do not produce measurable changes of the molecular extension along the pulling axis are not detected, and hence the energy landscape emerging from these studies is necessarily one-dimensional (Avdoshenko and Makarov, 2016). However, to circumvent this limitation an increasing number of solutions are emerging. For example, the attachment points of the DNA handles on the protein surface can be changed at will to pull the molecule along various reaction coordinates and assess different regions of its energy landscape (Elms et al., 2012; Heidarsson et al., 2012b). Alternatively, novel instrumentation designs with multiple optical traps can be employed that allow application of force along different pulling axes simultaneously (Dame et al., 2006). Otherwise, it is nowadays possible to use hybrid systems combining optical tweezers with single molecule Förster Resonance Energy Transfer (FRET) where the additional possibility of monitoring the distance between two or more residues suitably marked with fluorophores allows a multidimensional description of the molecular process under study (Sirinakis et al., 2012; Lee and Hohng, 2013).

## Simple Folding Mechanism Under Resting Conditions

The cellular environment contains a vast amount of different ions and most EF-hand proteins can bind other divalent ions besides calcium, most prominently Mg^2+^, which is kept relatively constant at ~5 mM in cells (Gifford et al., 2007; Grabarek, 2011). The Mg^2+^-bound state of EF hand proteins has many specific roles besides modulating Ca^2+^ binding and its properties are therefore of high interest (Zot and Potter, 1982; Wingard et al., 2005; Peshenko and Dizhoor, 2006; Aravind et al., 2008). In the case of NCS-1, the Mg^2+^-bound state seems to be the primary interacting conformation for many of its targets, such as the dopamine receptor D2 and P14Kb (Kabbani et al., 2002; Burgoyne et al., 2004) existing in equilibria with the apo and Ca^2+^-bound states. Elucidating the conformational dynamics of all these states (Burgoyne and Haynes, 2012) will help us understand better the molecular mechanisms mediating the biological functions of NCS-1.

At resting conditions in the cell, the two dominant forms of NCS-1 are the apo and Mg^2+^-bound states. In a recent publication, Naqvi et al. (2015) used optical tweezers to characterize at the single molecule level the conformational equilibria of these states for the non-myristoylated form of NCS-1. When they stretched and relaxed individual NCS-1 molecules in the absence of any divalent ions, they obtained force vs. extension traces characterized by reversible two-state fluctuations around 6.5 pN that originate from the folding and unfolding of the C-domain (Figure 1B). In fact, the N-domain of the apo form of NCS-1, as shown using deletion variants (Naqvi et al., 2015), is unstructured or loosely folded, and under tension it does not give rise to detectable transitions in the recorded traces. In contrast, under the same experimental conditions the C-domain is collapsed in a folded conformation that under tension displays a behavior that resembles that of molecular structures mainly stabilized by secondary interactions and weak tertiary contacts (Cecconi et al., 2005; Elms et al., 2012). Indeed, these data are also consistent with previous NMR studies showing little tertiary contacts in the apo form of NCS-1, and lack of stable globular structure (Cox et al., 1994; Aravind et al., 2008).

In the presence of 10 mM Mg^2+^ the behavior of NCS-1 changes drastically. Both the N- and C-domain acquire compact and mechanically resistant conformations that under tension loose and gain structure as separate units, through fully cooperative and sequential two-state transitions (Figure 1C; Naqvi et al., 2015). The C-domain is mechanically more resistant than the N-domain and unfolds at higher forces mirroring the observation from equilibrium chemical denaturation experiments (Aravind et al., 2008; Heidarsson et al., 2012a). After full mechanical denaturation of the protein and during relaxation of the applied force, the C-domain of NCS-1 is the first to fold, followed by the N-domain folding at lower forces. These sequential folding events are coordinated by Mg^2+^ binding to EF3 and EF2, respectively. The Mg^2+^-state of NCS-1 thus folds into its native state through a three-state process involving an intermediate state comprising a folded C-domain and an unstructured N-domain with ion occupancy only in EF3. The dimensions of the observed rips in the force vs. extension traces reveal more structuring of NCS-1 upon Mg^2+^ binding than previously suggested by NMR studies (Aravind et al., 2008), although the dynamical nature of the folded state is not revealed from the single molecule data.

## Increasing Folding Complexity Under Calcium Activating Conditions

In the presence of activating concentrations of calcium the folding process of non-myristoylated NCS-1 gets considerably more complex (Heidarsson et al., 2014). Under these experimental conditions, unfolded NCS-1 molecules fold back into their native states through a strict sequence of events coordinated by calcium binding (Figure 1D). Ca^2+^ first binds to EF3, triggering the collapse of the polypeptide chain (U state) into an on-pathway intermediate state I1; then EF4 binds a calcium ion, to induce the full folding of the C-domain (intermediate state I2). The last calcium ion finally binds to EF2 to fold the N-domain and thus the entire protein reaches its native state (N state). In this folding process, the C-domain always folds first and acts as “internal chaperone” for the correct folding of the N-domain. If the C-domain does not reach its native state, as in the knockout mutant NCS1^EF4^, the N-domain fails to fold correctly. This asymmetrical folding process that always starts with the folding of the C-domain probably reflects the asymmetrical structure of the protein, as further discussed below.

Each folding transition of NCS-1 is associated with a different structural change and energy cost. By monitoring in real time individual NCS-1 molecules fluctuating between different molecular conformations in constant force experiments, Heidarsson et al. (2013b) were able to characterize the kinetics and thermodynamics of the folding reactions of non-myristoylated NCS-1 and reconstruct the salient features of its energy landscape (Figures 1E,F). The results of these studies show how the folding transitions from U to I2 and from I1 to N are associated with large changes in molecular extensions but, at the same time, they are downhill reactions with no activation energy barriers. On the other hand, the transition from the intermediate state I2 to the intermediate state I1, which leads to the complete folding of the C-domain, involves a small compaction of the protein but is a barrier-limited reaction. Thus, folding of the C-domain into its native structure upon calcium binding to EF4 is the rate-limiting step of the entire folding process of NCS-1 at activating concentration of Ca^2+^. Interestingly, this step is affected by calcium concentration, which can open access to alternative and off-pathway folding trajectories.

## Pathologically High Calcium Concentrations Open Access to Misfolding Pathways

**Figure 2:**
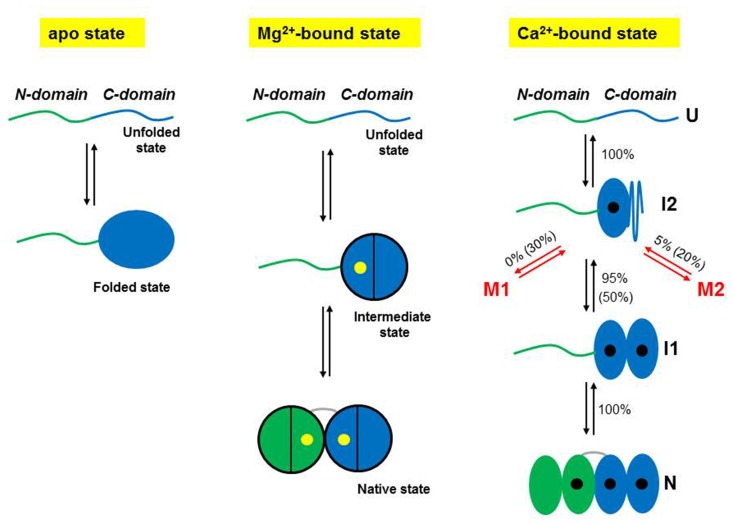
Schematic representations of the folding mechanisms of non-myristoylated NCS-1 in different ionic conditions. In the absence of divalent ions, the C-domain fluctuates between a folded and an unfolded state, while the N-domain remains unstructured or loosely folded. In the presence of Mg^2+^ (yellow dots), the divalent ions binds first to EF3, triggering the folding of the C-domain, and then to EF2 making the NCS-1 transit into its native state. Only two of the three active EF-hands can bind Mg^2+^. Under activating Ca^2+^ concentrations, NCS-1 folds into its native state through a four-state process involving the population of the intermediate states I1 and I2. First NCS-1 transits into I2 and then into I1 as Ca^2+^ (black dots) binds to EF3 and EF4, respectively. Finally Ca^2+^ binds to EF2 and the protein reaches N. At high calcium concentration (10 mM), only one molecule out of two follows this native folding pathway. Fifty percentage of the molecules folds into I2 and then takes non-productive pathways leading to misfolded conformations (M1 and M2). In contrast, at physiological calcium concentrations (0.5 μM), only 5% of the molecules misfold. Panels adapted from Heidarsson et al. (2014) and Naqvi et al. (2015).

Once an unfolded NCS-1 molecule has collapsed into the on-pathway intermediate state I2, it can either transit into I1 to eventually reach the native state, or take alternative pathways that lead to misfolded states (Heidarsson et al., 2014). The probability that the protein takes one pathway or the other depends on the calcium concentration. At physiologically relevant calcium concentration (0.5 μM) only 5% of the NCS-1 molecules misfold, but as the [Ca^2+^] increase, so does the misfolded population and at [Ca^2+^] of 10 mM one molecule out of two populates a pathway that leads to non-native structures. Two main misfolded states (M1 and M2) are populated by NCS-1, differing in extension and occupancy probability, with M1 being significantly populated only at high [Ca^2+^]. Figure 2 summarizes the folding and misfolding pathways of NCS-1 under different environmental conditions. Strikingly, misfolding reactions were observed only with wild-type NCS-1. Variants carrying disabled ion-binding sites never populate M1 or M2. These intriguing results suggest that the misfolding reactions observed in Heidarsson et al. (2014), stem from specific interactions that take place between properly folded EF hands. Inter-domain crosstalk thus plays a crucial role for the folding and misfolding of NCS-1, determining the final fate of the protein.

## Comparison to the Folding Network of Calmodulin

The folding and misfolding mechanisms of the other NCS family members and of other EF hand calcium binding proteins are still largely uncharacterized and the question remains: is it likely that the same principles of folding and misfolding can be inferred for these similar proteins? Other related proteins have been studied with regards to folding and stability using ensemble methods with varying degrees of details (Yamniuk et al., 2007; Suarez et al., 2008). However, to the best of our knowledge, the only EF hand protein whose conformational equilibria have been characterized in great detail at the single molecule level with optical tweezers is calmodulin (CaM; Stigler et al., 2011; Stigler and Rief, 2012), allowing for a direct comparison with NCS-1. The archetypical calcium binding protein CaM is characterized by a symmetrical structure with two almost identical domains, each binding two calcium ions, separated by a long and flexible α helix (Chattopadhyaya et al., 1992). The comparison between the folding network of NCS-1 and that of CaM indeed reveals many differences yet some similarities. The folding process of NCS-1 always starts with the U to I2 transition, indicating that only a single pathway is available on its energy landscape to initiate its journey to the native state. In contrast, unfolded CaM can initiate its folding process following three distinct pathways; one leading to an off-pathway intermediate (F_23_) with the EF hands 2 and 3 mispaired (16% probability), and the other two leading to either the on-pathway intermediate F_12_ (42% probability) or the on-pathway intermediate F_34_ (42% probability), with either the N- or the C-domain, respectively, fully folded. The folding process of CaM can thus start with equal probability from the N- or the C-domain, while that of NCS-1 always start from the C-domain and always through the same U to I2 transition. For CaM, F_12_ and F_34_ can then both transit into the native state, although with different probabilities as in 50% of the cases F_12_ takes a misfolding pathway leading to the off-pathway intermediate state F_123_, where EF3 is collapsed onto the folded N-domain in a non-native conformation. The native state of CaM can thus be reached from two distinct intermediate states through two different folding pathways, unlike the native state of NCS-1 that can be accessed only from I1. Moreover, the native unidimensional folding process of NCS-1 is characterized by a bottleneck where the protein can either proceed towards its native state by overcoming the large activation barrier separating I2 from I1 or take misfolding pathways in a calcium dependent manner. No bottleneck characterizes the folding network of CaM where the interconversions between the different molecular states are controlled by similar activation barriers. In addition, despite CaM folding rates and folding mechanism having been shown to be highly dependent on calcium concentration (Stigler and Rief, 2012), the population of CaM misfolding states have not yet been demonstrated to display such calcium dependence.

Where do the differences in the folding networks of CaM and NCS-1 originate from? The answer may lie in inter-domain interactions and the coupling free energies that are more developed in NCS-1. Proteins such as CaM have a flexible linker connecting their two domains, allowing a high degree of conformational variation between them and at the same time imposes separation of their folding funnels (Gifford et al., 2007; Kiran et al., 2017). For NCS-1, instead, a very short U-shaped linker of four residues (Ames et al., 2000) considerably limits the orientations of the two domains to face-to-face conformations, and the inter-domain interaction involves evolutionarily conserved residues pointing to a functional relevance of this interface. Additionally, NCS-1 is an asymmetrical protein as its N-domain has one active and one inactive EF hand, resulting in it being thermodynamically less stable than the C-domain (Heidarsson et al., 2012a), very unlike CaM where all EF-hands are active with similar calcium binding affinities (Masino et al., 2000). Moreover, NCS-1 carries a C-terminal tail that has an N-domain stabilizing effect (Heidarsson et al., 2012a) and this effect may only become functional, once the C-domain is folded. Thus, the sequential folding observed for NCS-1 could be the result of its intricate structural features and its biological function as a sensor. More studies on related NCS proteins will undoubtedly help clarify these issues. Nevertheless, given the complex folding networks of CaM and NCS-1, and the frequent occurrence of misfolded states in multi-domain proteins (Han et al., 2007; Borgia et al., 2011, 2015) we predict that misfolding is likely to occur in similar calcium binding proteins.

## Conclusions and Future Perspectives

We have outlined how the Ca^2+^-bound, Mg^2+^-bound and apo forms of the non-myristoylated NCS-1 have been investigated at the single molecule level using optical tweezers. We have illustrated the rugged and multistate energy landscape arising from these studies, which underlays the calcium-dependent folding and misfolding trajectories of NCS-1. We have also discussed the importance that the conformational sensitivity of NCS-1 might have for its calcium sensing actions. Finally, we have made comparison to the very similar calcium binding EF hand protein CaM and have highlighted differences and similarities between the folding networks of the two proteins, which are likely to have arisen due to their different structural and functional features across the domains.

For NCS-1, the calcium-dependent modulation of misfolding pathways is highly interesting and suggests an important link between the conformational space, calcium dysregulation, and neurodegeneration. Interestingly, sustained elevated levels of free Ca^2+^ have been associated with aging cells and linked to the development of major neurological diseases such as bipolar disorder and Alzheimer’s (Toescu and Vreugdenhil, 2010; Berridge, 2012). In some neurological diseases, such as bipolar disorder and schizophrenia, NCS-1 is up-regulated in the pre-frontal cortex of patients (Koh et al., 2003). A compelling hypothesis would be that the upregulation is a response to a loss of protein function caused by calcium-induced misfolding. Studies in cell, focusing directly on the calcium effects on NCS-1 expression levels might reveal whether there indeed is a specific phenotype associated with NCS-1 misfolding, and potentially also of other members of the family.

The behavior of NCS-1 outlined in this review article may also help rationalize how NCS-1 is able to have such promiscuous interactions with over 20 reported binding partners (Burgoyne, 2007) despite a highly folded and globular structure. A certain degree of flexibility must thus be required to accommodate this list of diverse ligands such as dopamine receptors, various ion-channels, glial cell line-derived neurotrophic factor as well as membranes and their constituents. The many conformational states that were uncovered in the articles discussed above may reflect a significant flexibility of NCS-1, which is needed for it to act as a molecular hub (Heidarsson et al., 2013b, 2014; Naqvi et al., 2015). These properties may be even further expanded including its N-terminal myristoylation, which leads to localization and broader range of interactors. How this lipidic modification changes the molecular landscape of NCS-1 remains to be addressed in detail. The single molecule approach provided by optical tweezers can potentially be used to probe the interaction of NCS-1 with different ligands as well as its modulation by posttranslational modification, and help uncover the connection between conformational states, ligand recognition and disease states. Indeed, the two EF hand proteins for which detailed insight into their single-molecule folding networks exist (Stigler et al., 2011; Heidarsson et al., 2014) have revealed that single-molecule approaches are key to their studies and enable the extraction of important insight into structures as well as function and dysfunction.

## Author Contributions

DC designed and wrote the article; carried out bibliographic research. BK assisted in structural details and biological aspects, and contributed in the final revisions of the manuscript. PH designed and wrote the article. CC designed and wrote the article; prepared the figures. All authors have provided final approval of the version to be published.

## Conflict of Interest Statement

The authors declare that the research was conducted in the absence of any commercial or financial relationships that could be construed as a potential conflict of interest.

## References

[B1] Alemany A. Rey-Serra B. Frutos S. Cecconi C. Ritort F. (2016). Mechanical folding and unfolding of protein barnase at the single-molecule level. Biophys. J. 110, 63–74. 10.1016/j.bpj.2015.11.015 26745410 PMC4825109

[B2] Ames J. B. Hendricks K. B. Strahl T. Huttner I. G. Hamasaki N. Thorner J. (2000). Structure and calcium-binding properties of Frq1, a novel calcium sensor in the yeast *Saccharomyces cerevisiae*. Biochemistry 39, 12149–12161. 10.1021/bi0012890 11015193

[B3] Aravind P. Chandra K. Reddy P. P. Jeromin A. Chary K. Sharma Y. (2008). Regulatory and structural EF-hand motifs of neuronal calcium sensor-1: Mg^2+^ modulates Ca^2+^ binding, Ca^2+^-induced conformational changes, and equilibrium unfolding transitions. J. Mol. Biol. 376, 1100–1115. 10.1016/j.jmb.2007.12.033 18199453

[B4] Avdoshenko S. M. Makarov D. E. (2016). Reaction coordinates and pathways of mechanochemical transformations. J. Phys. Chem. B 120, 1537–1545. 10.1021/acs.jpcb.5b07613 26401617

[B5] Bai J. He F. Novikova S. I. Undie A. S. Dracheva S. Haroutunian V.. (2004). Abnormalities in the dopamine system in schizophrenia may lie in altered levels of dopamine receptor-interacting proteins. Biol. Psychiatry 56, 427–440. 10.1016/j.biopsych.2004.06.022 15364041

[B6] Batey S. Nickson A. A. Clarke J. (2008). Studying the folding of multidomain proteins. HFSP J. 2, 365–377. 10.2976/1.2991513 19436439 PMC2645590

[B7] Berridge M. J. (2012). Calcium signalling remodelling and disease. Biochem. Soc. Trans. 40, 297–309. 10.1042/bst20110766 22435804

[B9] Borgia M. B. Borgia A. Best R. B. Steward A. Nettels D. Wunderlich B.. (2011). Single-molecule fluorescence reveals sequence-specific misfolding in multidomain proteins. Nature 474, 662–665. 10.1038/nature10099 21623368 PMC3160465

[B8] Borgia A. Kemplen K. R. Borgia M. B. Soranno A. Shammas S. Wunderlich B.. (2015). Transient misfolding dominates multidomain protein folding. Nat. Commun. 6:8861. 10.1038/ncomms9861 26572969 PMC4660218

[B10] Bourne Y. Dannenberg J. Pollmann V. Marchot P. Pongs O. (2001). Immunocytochemical localization and crystal structure of human frequenin (neuronal calcium sensor 1). J. Biol. Chem. 276, 11949–11955. 10.1074/jbc.m009373200 11092894

[B11] Burgoyne R. D. (2007). Neuronal calcium sensor proteins: generating diversity in neuronal Ca^2+^ signalling. Nat. Rev. Neurosci. 8, 182–193. 10.1038/nrn2093 17311005 PMC1887812

[B12] Burgoyne R. D. Haynes L. P. (2012). Understanding the physiological roles of the neuronal calcium sensor proteins. Mol. Brain 5:2. 10.1186/1756-6606-5-2 22269068 PMC3271974

[B13] Burgoyne R. D. O’Callaghan D. W. Hasdemir B. Haynes L. P. Tepikin A. V. (2004). Neuronal Ca^2+^-sensor proteins: multitalented regulators of neuronal function. Trends Neurosci. 27, 203–209. 10.1016/j.tins.2004.01.010 15046879

[B14] Caldarini M. Sonar P. Valpapuram I. Tavella D. Volonté C. Pandini V.. (2014). The complex folding behavior of HIV-1-protease monomer revealed by optical-tweezer single-molecule experiments and molecular dynamics simulations. Biophys. Chem. 195, 32–42. 10.1016/j.bpc.2014.08.001 25194276

[B15] Cecconi C. Shank E. A. Bustamante C. Marqusee S. (2005). Direct observation of the three-state folding of a single protein molecule. Science 309, 2057–2060. 10.1126/science.1116702 16179479

[B16] Chattopadhyaya R. Meador W. E. Means A. R. Quiocho F. A. (1992). Calmodulin structure refined at 1.7 angstrom resolution. J. Mol. Biol. 228, 1177–1192. 10.1016/0022-2836(92)90324-D 1474585

[B17] Chodera J. D. Elms P. Noé F. Keller B. Kaiser C. M. Ewall-Wice A. (2011). Bayesian hidden Markov model analysis of single-molecule force spectroscopy: characterizing kinetics under measurement uncertainty. arXiv:1108.1430 [preprint].

[B18] Cox J. A. Durussel I. Comte M. Nef S. Nef P. Lenz S. E.. (1994). Cation binding and conformational changes in VILIP and NCS-1, two neuron-specific calcium-binding proteins. J. Biol. Chem. 269, 32807–32813. 7806504

[B19] Dame R. T. Noom M. C. Wuite G. J. L. (2006). Bacterial chromatin organization by H-NS protein unravelled using dual DNA manipulation. Nature 444, 387–390. 10.1038/nature05283 17108966

[B21] Elms P. J. Chodera J. D. Bustamante C. Marqusee S. (2012). The molten globule state is unusually deformable under mechanical force. Proc. Natl. Acad. Sci. U S A 109, 3796–3801. 10.1073/pnas.1115519109 22355138 PMC3309780

[B22] Gebhardt J. C. M. Bornschlögla T. Rief M. (2010). Full distance-resolved folding energy landscape of one single protein molecule. Proc. Natl. Acad. Sci. U S A 107, 2013–2018. 10.1073/pnas.0909854107 20133846 PMC2836620

[B23] Gifford J. L. Walsh M. P. Vogel H. J. (2007). Structures and metal-ion-binding properties of the Ca^2+^-binding helix-loop-helix EF-hand motifs. Biochem. J. 405, 199–221. 10.1042/bj20070255 17590154

[B24] Grabarek Z. (2011). Insights into modulation of calcium signaling by magnesium in calmodulin, troponin C and related EF-hand proteins. Biochim. Biophys. Acta 1813, 913–921. 10.1016/j.bbamcr.2011.01.017 21262274 PMC3078997

[B25] Guo W. Malin S. A. Johns D. C. Jeromin A. Nerbonne J. M. (2002). Modulation of Kv4-encoded K^+^ currents in the mammalian myocardium by neuronal calcium sensor-1. J. Biol. Chem. 277, 26436–26443. 10.1074/jbc.m201431200 11994284

[B26] Han J. H. Batey S. Nickson A. A. Teichmann S. A. Clarke J. (2007). The folding and evolution of multidomain proteins. Nat. Rev. Mol. Cell Biol. 8, 319–330. 10.1038/nrm2144 17356578

[B27] Handley M. T. W. Lian L. Y. Haynes L. P. Burgoyne R. D. (2010). Structural and functional deficits in a neuronal calcium sensor-1 mutant identified in a case of autistic spectrum disorder. PLoS One 5:e10534. 10.1371/journal.pone.0010534 20479890 PMC2866544

[B28] Heidarsson P. O. Bjerrum-Bohr I. J. Jensen G. A. Pongs O. Finn B. E. Poulsen F. M.. (2012a). The C-terminal tail of human neuronal calcium sensor 1 regulates the conformational stability of the Ca^2+^-activated state. J. Mol. Biol. 417, 51–64. 10.1016/j.jmb.2011.12.049 22227393

[B32] Heidarsson P. O. Valpapuram I. Camilloni C. Imparato A. Tiana G. Poulsen F. M.. (2012b). A highly compliant protein native state with a spontaneous-like mechanical unfolding pathway. J. Am. Chem. Soc. 134, 17068–17075. 10.1021/ja305862m 23004011

[B29] Heidarsson P. O. Naqvi M. M. Otazo M. R. Mossa A. Kragelund B. B. Cecconi C. (2014). Direct single-molecule observation of calcium-dependent misfolding in human neuronal calcium sensor-1. Proc. Natl. Acad. Sci. U S A 111, 13069–13074. 10.1073/pnas.1401065111 25157171 PMC4246975

[B30] Heidarsson P. O. Naqvi M. M. Sonar P. Valpapuram I. Cecconi C. (2013a). Conformational dynamics of single protein molecules studied by direct mechanical manipulation. Adv. Protein Chem. Struct Biol. 92, 93–133. 10.1016/b978-0-12-411636-8.00003-1 23954100

[B31] Heidarsson P. O. Otazo M. R. Bellucci L. Mossa A. Imparato A. Paci E.. (2013b). Single-molecule folding mechanism of an EF-hand neuronal calcium sensor. Structure 21, 1812–1821. 10.1016/j.str.2013.07.022 24012477

[B33] Jahn M. Buchner J. Hugel T. Rief M. (2016). Folding and assembly of the large molecular machine Hsp90 studied in single-molecule experiments. Proc. Natl. Acad. Sci. U S A 113, 1232–1237. 10.1073/pnas.1518827113 26787848 PMC4747692

[B34] Jahn M. Tych K. Girstmair H. Steinmassl M. Hugel T. Buchner J.. (2018). Folding and domain interactions of three orthologs of Hsp90 studied by single-molecule force spectroscopy. Structure 26, 96.e4–105.e4. 10.1016/j.str.2017.11.023 29276035

[B35] Kabbani N. Negyessy L. Lin R. Goldman-Rakic P. Levenson R. (2002). Interaction with neuronal calcium sensor NCS-1 mediates desensitization of the D2 dopamine receptor. J. Neurosci. 22, 8476–8486. 10.1523/JNEUROSCI.22-19-08476.2002 12351722 PMC6757796

[B36] Kiran U. Regur P. Kreutz M. R. Sharma Y. Chakraborty A. (2017). Intermotif communication induces hierarchical Ca^2+^ filling of caldendrin. Biochemistry 56, 2467–2476. 10.1021/acs.biochem.7b00132 28437073

[B37] Koh P. O. Undie A. S. Kabbani N. Levenson R. Goldman-Rakic P. S. Lidow M. S. (2003). Up-regulation of neuronal calcium sensor-1 (NCS-1) in the prefrontal cortex of schizophrenic and bipolar patients. Proc. Natl. Acad. Sci. U S A 100, 313–317. 10.1073/pnas.232693499 12496348 PMC140961

[B38] Lee S. Hohng S. (2013). An optical trap combined with three-color FRET. J. Am. Chem. Soc. 135, 18260–18263. 10.1021/ja408767p 24256200

[B39] Masino L. Martin S. R. Bayley P. M. (2000). Ligand binding and thermodynamic stability of a multidomain protein, calmodulin. Protein Sci. 9, 1519–1529. 10.1110/ps.9.8.1519 10975573 PMC2144730

[B40] McFerran B. W. Weiss J. L. Burgoyne R. D. (1999). Neuronal Ca^2+^ sensor 1. Characterization of the myristoylated protein, its cellular effects in permeabilized adrenal chromaffin cells, Ca^2+^-independent membrane association and interaction with binding proteins, suggesting a role in rapid Ca^2+^ signal transduction. J. Biol. Chem. 274, 30258–30265. 10.1074/jbc.274.42.30258 10514519

[B41] Nakamura T. Y. Pountney D. J. Ozaita A. Nandi S. Ueda S. Rudy B.. (2001). A role for frequenin, a Ca^2+^-binding protein, as a regulator of Kv4 K^+^-currents. Proc. Natl. Acad. Sci. U S A 98, 12808–12813. 10.1073/pnas.221168498 11606724 PMC60135

[B42] Naqvi M. M. Heidarsson P. O. Otazo M. R. Mossa A. Kragelund B. B. Cecconi C. (2015). Single-molecule folding mechanisms of the apo-and Mg^2+^-bound states of human neuronal calcium sensor-1. Biophys. J. 109, 113–123. 10.1016/j.bpj.2015.05.028 26153708 PMC4572569

[B43] Pavlowsky A. Gianfelice A. Pallotto M. Zanchi A. Vara H. Khelfaoui M.. (2010). A postsynaptic signaling pathway that may account for the cognitive defect due to IL1RAPL1 mutation. Curr. Biol. 20, 103–115. 10.1016/j.cub.2009.12.030 20096586

[B44] Peshenko I. V. Dizhoor A. M. (2006). Ca^2+^ and Mg^2+^ binding properties of GCAP-1 evidence that Mg^2+^-bound form is the physiological activator of photoreceptor guanylyl cyclase. J. Biol. Chem. 281, 23830–23841. 10.1074/jbc.M600257200 16793776

[B45] Pongs O. Lindemeier J. Zhu X. R. Theil T. Engelkamp D. Krah-Jentgens I.. (1993). Frequenin—a novel calcium-binding protein that modulates synaptic efficacy in the drosophila nervous-system. Neuron 11, 15–28. 10.1016/0896-6273(93)90267-u 8101711

[B46] Rabiner L. R. (1989). A tutorial on hidden markov-models and selected applications in speech recognition. Proc. IEEE 77, 257–286. 10.1109/5.18626

[B47] Reyes-Bermudez A. Miller D. J. Sprungala S. (2012). The neuronal calcium sensor protein acrocalcin: a potential target of calmodulin regulation during development in the coral *Acropora millepora*. PLoS One 7:e51689. 10.1371/journal.pone.0051689 23284743 PMC3524228

[B48] Rivosecchi R. Pongs O. Theil T. Mallart A. (1994). Implication of frequenin in the facilitation of transmitter release in *Drosophila*. J. Physiol. 474, 223–232. 10.1113/jphysiol.1994.sp020015 7911829 PMC1160311

[B49] Scannevin R. H. Wang K. Jow F. Megules J. Kopsco D. C. Edris W.. (2004). Two N-terminal domains of Kv4 K^+^ channels regulate binding to and modulation by KChIP1. Neuron 41, 587–598. 10.1016/s0896-6273(04)00049-2 14980207

[B50] Shank E. A. Cecconi C. Dill J. W. Marqusee S. Bustamante C. (2010). The folding cooperativity of a protein is controlled by its chain topology. Nature 465, 637–640. 10.1038/nature09021 20495548 PMC2911970

[B51] Sirinakis G. Ren Y. X. Gao Y. Xi Z. Q. Zhang Y. L. (2012). Combined versatile high-resolution optical tweezers and single-molecule fluorescence microscopy. Rev. Sci. Instrum. 83:093708. 10.1063/1.4752190 23020384 PMC3465359

[B52] Smith S. B. Cui Y. J. Bustamante C. (2003). “Optical-trap force transducer that operates by direct measurement of light momentum,” in Biophotonics, Pt B, eds Marriott G. Parker I. (San Diego, CA: Elsevier Academic Press Inc.), 134–162. 10.1016/s0076-6879(03)61009-8 12624910

[B53] Stigler J. Rief M. (2012). Calcium-dependent folding of single calmodulin molecules. Proc. Natl. Acad. Sci. U S A 109, 17814–17819. 10.1073/pnas.1201801109 22753517 PMC3497792

[B54] Stigler J. Ziegler F. Gieseke A. Gebhardt J. C. M. Rief M. (2011). The complex folding network of single calmodulin molecules. Science 334, 512–516. 10.1126/science.1207598 22034433

[B55] Strahl T. Huttner I. G. Lusin J. D. Osawa M. King D. Thorner J.. (2007). Structural insights into activation of phosphatidylinositol 4-kinase (Pik1) by yeast frequenin (Frq1). J. Biol. Chem. 282, 30949–30959. 10.1074/jbc.m705499200 17720810

[B56] Suarez M. C. Rocha C. B. Sorenson M. M. Silva J. L. Foguel D. (2008). Free-energy linkage between folding and calcium binding in EF-hand proteins. Biophys. J. 95, 4820–4828. 10.1529/biophysj.108.135715 18689462 PMC2576379

[B57] Toescu E. C. Vreugdenhil M. (2010). Calcium and normal brain ageing. Cell calcium 47, 158–164. 10.1016/j.ceca.2009.11.013 20045187

[B58] Tsujimoto T. Jeromin A. Saitoh N. Roder J. C. Takahashi T. (2002). Neuronal calcium sensor 1 and activity-dependent facilitation of P/Q-type calcium currents at presynaptic nerve terminals. Science 295, 2276–2279. 10.1126/science.1068278 11910115

[B59] Weiss J. L. Archer D. A. Burgoyne R. D. (2000). Neuronal Ca^2+^ sensor-1/frequenin functions in an autocrine pathway regulating Ca^2+^ channels in bovine adrenal chromaffin cells. J. Biol. Chem. 275, 40082–40087. 10.1074/jbc.M008603200 11006299

[B60] Weiss J. L. Hui H. Burgoyne R. D. (2010). Neuronal calcium sensor-1 regulation of calcium channels, secretion, and neuronal outgrowth. Cell. Mol. Neurobiol. 30, 1283–1292. 10.1007/s10571-010-9588-7 21104311 PMC11498851

[B61] Wingard J. N. Chan J. Bosanac I. Haeseleer F. Palczweski K. Ikura M.. (2005). Structural analysis of Mg^2+^ and Ca^2+^ binding to calcium-binding protein 1 (CaBP1), a neuron-specific regulator of calcium channels. J. Biol. Chem. 280, 37461–37470. 10.1074/jbc.M508541200 16147998 PMC1470661

[B62] Wruck F. Avellaneda M. J. Koers E. J. Minde D. P. Mayer M. P. Kramer G.. (2018). Protein folding mediated by trigger factor and Hsp70: new insights from Single-Molecule approaches. J. Mol. Biol. 430, 438–449. 10.1016/j.jmb.2017.09.004 28911846

[B63] Xu A. J. Springer T. A. (2013). Mechanisms by which von Willebrand disease mutations destabilize the A2 domain. J. Biol. Chem. 288, 6317–6324. 10.1074/jbc.m112.422618 23322777 PMC3585066

[B64] Yamniuk A. P. Silver D. M. Anderson K. L. Martin S. R. Vogel H. J. (2007). Domain stability and metal-induced folding of calcium- and integrin-binding protein 1. Biochemistry 46, 7088–7098. 10.1021/bi700200z 17516631

[B65] Yu H. Liu X. Neupane K. Gupta A. N. Brigley A. M. Solanki A.. (2012). Direct observation of multiple misfolding pathways in a single prion protein molecule. Proc. Natl. Acad. Sci. U S A 109, 5283–5288. 10.1073/pnas.1107736109 22421432 PMC3325692

[B66] Zhang Y. (2017). Energetics, kinetics, and pathway of SNARE folding and assembly revealed by optical tweezers. Protein Sci. 26, 1252–1265. 10.1002/pro.3116 28097727 PMC5477538

[B67] Zhou W. Qian Y. Kunjilwar K. Pfaffinger P. J. Choe S. (2004). Structural insights into the functional interaction of KChIP1 with Shal-type K^+^ channels. Neuron 41, 573–586. 10.1016/s0896-6273(04)00045-5 14980206

[B68] Zot H. Potter J. (1982). A structural role for the Ca^2+^-Mg^2+^ sites on troponin C in the regulation of muscle contraction. Preparation and properties of troponin C depleted myofibrils. J. Biol. Chem. 257, 7678–7683. 6211445

